# A new perspective is needed for positive selection of germinal center B cells with higher-affinity B cell receptors

**DOI:** 10.1038/s41423-021-00823-4

**Published:** 2022-01-07

**Authors:** Timo Gaber, Frank Buttgereit

**Affiliations:** Department of Rheumatology and Clinical Immunology, Charité – Universitätsmedizin Berlin, corporate member of Freie Universität Berlin and Humboldt Universität zu Berlin, Berlin, Germany

**Keywords:** B cells, B-cell receptor

**Fig. 1:**
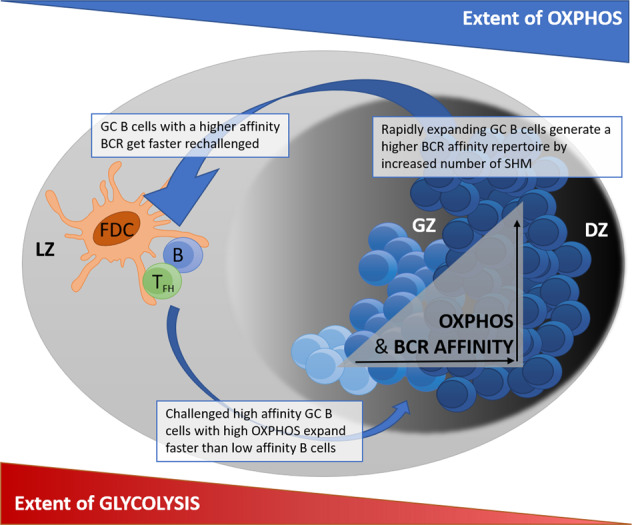
Schematic illustration of a germinal center (GC) displaying the spatial distribution and metabolic programs of GC B cells during BCR affinity maturation. OXPHOS oxidative phosphorylation, LZ light zone, GZ gray zone, DZ dark zone, follicular dendritic cells (FDCs) = orange; follicular T helper cells (T_FH_) cells = green; germinal center (GC) B cells = blue (increasing BCR affinity = increasing blue intensity), BCR B cell receptor

In a recent issue of Nature Immunology, Chen et al. identified differential expression signatures of metabolic programs within the germinal center (GC) compartment to distinguish GC B cells from different zones [1]. Furthermore, they identified an important role of oxidative phosphorylation (OXPHOS) in the process of positive selection of B cells with higher-affinity B cell receptors (BCRs) in GCs. GCs are inducible secondary lymphoid microanatomical structures that provide niches for B cells to capture and present antigens in the light zone (LZ) and to undergo clonal expansion and BCR somatic hypermutation (SHM) in the dark zone (DZ) Fig. 1. Alternating migration of activated GC B cells between the LZ and DZ is assumed to result in positive selection of clones with higher-affinity B cell antigen receptors. These clones are characterized by accelerated cell division, suggesting a genomic program activated by BCR and CD40 signaling [2]. Although GC B cells of the LZ have been suggested to rely primarily on glycolysis due to the hypoxic nature of the LZ niche, isolated GC B cells demonstrated an oxidative phenotype in vitro [3]. Chen et al. recently reported that GC B cells require OXPHOS activity not only for their clonal expansion but also, and at least as importantly, for efficient positive selection of high-affinity BCR-expressing GC B cell clones in the DZ. The researchers also first described a link between switching of the GC B cell metabolic profile, cell cycle control, cell expansion, and positive selection of cells with high-affinity B cell receptors. These observations clearly underline that cellular energy metabolism is an important part of the background machinery that ensures the proper functioning of immune cells (here, GC B cells)–a view that is more than 20 years old [4].

Antigen challenge of B cells with the help of follicular T helper cells in GCs results in clonal expansion, expression of high-affinity antibodies, and antibody class switching generating B cell memory. Under the well-demonstrated hypoxic LZ conditions, HIF signaling is induced in GC B cells [3]. This leads to increased glycolysis, reduced B cell expansion, impaired class switching to the IgG2c antibody isotype, and enhanced B cell death. Of note, constitutively active HIF-1α signaling has been demonstrated to increase B cell expansion, decrease the number of antigen-specific GC B cells, and impair the generation of high-affinity IgG antibodies [5]. The formation and maintenance of GC-B cells thus depend on HIF-mediated enhancement of glycolysis and mitochondrial biogenesis for growth and proliferation. In line with this, reduced glucose uptake by deletion of glucose transporter 1 decreases B cell proliferation and impairs antibody production [5]. The data presented by Chen et al. support an iterative process of BCR affinity maturation induced by migration of GC B cells back and forth between the LZ via the gray zone (GZ) and DZ with the need for expression of different metabolic profiles (Fig. 1). To gain these important fundamental insights into GC B cell biology, the authors cleverly coupled single-cell RNA sequencing (scRNA-seq) with tracking of cells with positively selected BCR mutations.

For GC B cell profiling, the authors used the classical T cell-dependent antigen 4-hydroxy-3-nitrophenylacetyl (NP) conjugated with keyhole limpet hemocyanin (KLH) [6]. The authors immunized mice with NP-KLH and induced a GC B cell response dominated by clones encoding the V_H_186.2 variable heavy chain gene segment. V_H_186.2 is highly susceptible to a W33L amino acid substitution due to SHM, which results in high-affinity BCR-positive GC B cells. Using this approach, the authors were able to identify, separate and collect the high-affinity NP-specific GC B cells that acquired the W33L mutation from their counterparts that did not have the mutation and thus low-affinity BCRs for analysis to distinguish their genomic states [1].

Based on scRNA-seq technology, the authors stratified GC B cells into 6 clusters: 1 cluster representing genes associated with antigen capture, BCR signaling and interaction with T_FH_ cells for GC B cells located in the LZ; 1 cluster of genes mainly associated with resting B cell states; 3 clusters displaying increased amounts of transcripts involved in DNA replication, chromatin segregation and cell division, representing GC B cells in successive phases of the cell cycle, namely, G1 → S, S → G2 and G2 → M for each cluster; and 1 cluster with low mitotic activity expressing higher amounts of DZ but lower amounts of LZ genes [1]. The authors then used the so-called topic modeling technique, in which cells are depicted in the form of zone markers and a weighted admixture of genomic programs (topics) [7]. This approach enabled the identification of previously unknown metabolic programs with different expression dynamics of OXPHOS and glycolysis in GC B cells, suggesting that DZ GC B cells undergoing rapid mitosis preferentially use OXPHOS and fatty acid oxidation (FAO) [8]. Finally, coupled analysis of SHM (*Igh* variable gene mutation profiling) and the transcriptomes of NP-specific GC B cells implicated increased OXPHOS in the positive selection of GC B cell clones that acquire a SHM-mediated higher-affinity BCR. Using the same approach, the authors confirmed their results of an increased OXPHOS program in dominant GC B cells that responded to the complex OVA antigen. As a prerequisite for this approach, the authors first demonstrated that a mutation in the IgHV1-26*01 gene with a S59T substitution was undergoing positive selection, generating OVA-binding high-affinity BCRs. Again, GC B cell clones with this positively selected mutation (S59T) had higher expression of OXPHOS pathway genes than their control counterparts [1].

Since loss of *Cox10*, which encodes an essential protein for the assembly of terminal complex IV of the electron transport chain, significantly impairs OXPHOS activity in B cell lines, the authors immunized mice with B cell-specific deletion of the *Cox10* gene (*Cox10*^*fl/fl*^ *Aicda*^*+/cre*^) using NP-KLH. Using this loss-of-function approach, the authors have impressively proven that GC B cells require OXPHOS activity not only for their clonal expansion but also, importantly, for efficient positive selection. Conversely, enhancement of OXPHOS with oltipraz, a substance that significantly increases the oxygen consumption rate (OCR) of in vitro GC B cells, promoted affinity maturation in vivo in the context of a GC response induced by NP-KLH immunization.

In summary, both the framework used and the results reported by Chen et al. are of great interest for other research questions since a similar approach may be useful to identify transcriptional and metabolic features of immunoprotective, autoreactive, or tolerant B cell clones in immunological disorders such as rheumatoid arthritis or systemic lupus erythematosus. Both diseases have been attributed to cellular metabolic abnormalities, such as those observed in T cells and autoantibody production. Although the publication by Chen et al. reports on carefully conducted experiments with clear and novel conclusions, it also has limitations. First, it needs to be shown that the findings obtained in murine models can also be confirmed for the positive selection of human GC B cells with high-affinity BCRs. Second, the chemical augmentation of OXPHOS with oltipraz for the induction of affinity maturation in vivo may also impact other cell types. Oltipraz treatment of mice does not only affect GC B cells of the DZ, where GC B cell proliferation and affinity maturation rely on OXPHOS, and may also interfere with GC B cell activation or the expansion of T_FH_ cells. Coupling histological data, scRNA-seq data, and data from tracking cells with positively selected BCR mutations using spatial RNA sequencing (spRNA-seq) may clearly visualize the three-dimensional iterative process of zone-specific high-affinity BCR maturation of GC B cells [9].

Finally, it remains elusive whether BCR-mediated induction of either cell cycle-induced OXPHOS or OXPHOS-induced cell cycle processes result in clonal expansion and enhancement of positive selection in a “top-down” control mechanism of metabolic reprogramming. Moreover, the study does not rule out the possibility that the induction of OXPHOS by an increase in oxygen availability in the DZ leads to an increase in the cell cycle of only BCR-activated cells in “bottom-up” metabolic signaling. Currently, no precise and comprehensive concept on how the mechanistic cascade works to increase the OXPHOS program in GC B cells for high-affinity BCR maturation exists. Of note, the latter would be urgently needed to further and decisively target transcriptional and metabolic features of, e.g., autoreactive B cell clones in immunological disorders.
